# COP9 signalosome subunit 5 regulates cancer metastasis by deubiquitinating SNAIL

**DOI:** 10.18632/oncotarget.25060

**Published:** 2018-04-17

**Authors:** Kensuke Watanabe, Satoru Yokoyama, Naoki Kaneto, Takashi Hori, Yusuke Iwakami, Shinichiro Kato, Yoshihiro Hayakawa, Hiroaki Sakurai, Junya Fukuoka, Ikuo Saiki

**Affiliations:** ^1^ Division of Pathogenic Biochemistry, Institute of Natural Medicine, University of Toyama, Toyama 930-0194, Japan; ^2^ Department of Diagnostic Pathology, Toyama University Hospital, Toyama 930-0194, Japan; ^3^ Department of Cancer Cell Biology, Graduate School of Medicine and Pharmaceutical Sciences, University of Toyama, Toyama 930-0194, Japan; ^4^ Department of Pathology, Nagasaki University Graduate School of Biomedical Sciences, Nagasaki 852-8501, Japan

**Keywords:** SNAIL, metastasis, COPS5, deubiquitination, lung cancer

## Abstract

Cancer metastasis is a major cause of mortality in cancer patients. The transcription factor SNAIL plays an important role in cancer metastasis and progression, and its expression is tightly regulated by the ubiquitin-proteasome system through the balance between ubiquitin ligases and deubiquitinating enzymes. While several ubiquitin ligases of SNAIL have been identified, it is not yet clear regarding deubiquitinating enzyme. In this study, we identified COP9 signalosome subunit 5 (COPS5) as a deubiquitinating enzyme of SNAIL by using siRNA library screening. COPS5 downregulation significantly reduced the expression of SNAIL and impaired the metastatic potential of lung cancer cells both *in vitro* and *in vivo*. Importantly, we demonstrated that COPS5 binds to SNAIL and stabilizes its expression by deubiquitination. Furthermore, we observed the positive correlation between COPS5 and SNAIL expression in the clinical tissue samples of lung adenocarcinomas by using tissue microarray analysis. These findings provide strong evidence that COPS5 can be a new therapeutic target for cancer metastasis as a deubiquitinating enzyme of SNAIL.

## INTRODUCTION

The critical characteristics of cancers are the ability to invade surrounding tissues and metastasize to distal tissues, which is the major cause of mortality in cancer patients [1]. Cancer metastasis consists of several steps: intravasation, attachment to a vessel, extravasation, angiogenesis, and growth in distal tissues [2]. The migration ability or invasiveness of cancer cells plays a critical role during metastasis.

SNAIL is a transcription factor that regulates the migration ability in some cancer types, including lung and pancreas [3]. Recent studies suggest that SNAIL has a much broader impact on cancer progression such as the induction of epithelial-to-mesenchymal transition [3], an enhancement of recruitment of macrophages [4], an induction of tumor-initiating properties [5], a suppression of host immune surveillance [6], drug resistance [7], and cancer metabolism [8]. Given that SNAIL has multiple functions in cancer progression, it is important to understand the regulatory mechanisms for SNAIL in cancers, resulting in the acquisition of a new therapeutic strategy for cancer progression by targeting SNAIL.

SNAIL has been transcriptionally regulated by some pathways, including transforming growth factor-β, NOTCH, and WNT pathways, reactive oxygen species, and hypoxic stress [9]. In addition to transcriptional regulation, SNAIL has been regulated post-translationally through the ubiquitin-proteasome system. Protein ubiquitination is an essential modification and is tightly regulated by the balance between ubiquitination and deubiquitination. In the case of SNAIL, at least four E3 ubiquitin ligases, FBXO11 [10], FBXW1 [11], FBXL5 [12], and FBXL14 [13, 14], have been identified as the responsible enzymes for SNAIL ubiquitination and degradation. In contrast, few deubiquitinating enzymes (DUBs) for SNAIL has been identified [15], though approximately 100 putative DUBs are known in human [16]. A protein ubiquitination has been classified into three groups, K48-linked, K63-linked, and M1-linked polyubiquitination. K48-linked and K63-linked polyubiquitination have been mainly implicated in proteasomal degradation or endosomal sorting/lysosomal targeting, respectively. Even in DUBs, their substrates have been shown to have specificities to K48-, K63-, or M1-linked polyubiquitination.

In this study, we identify COP9 signalosome subunit 5 (COPS5), one of the DUBs, as a deubiquitinating enzyme of SNAIL by HA-based siRNA screening. COPS5 knock-down shows a significant inhibition of invasiveness *in vitro* and also lung metastasis *in vivo*. By functional studies, we determine the COPS5-mediated stabilization of SNAIL through its deubiquitination. Furthermore, the positive correlation between COPS5 and SNAIL expression is observed in the clinical tissue samples of lung adenocarcinomas using tissue microarray analysis. Collectively, COPS5 could be a good molecular target for cancer metastasis through SNAIL degradation.

## RESULTS

### Deubiquitinating enzymes regulate SNAIL expression and invasion in cancer cells

**Figure 1:**
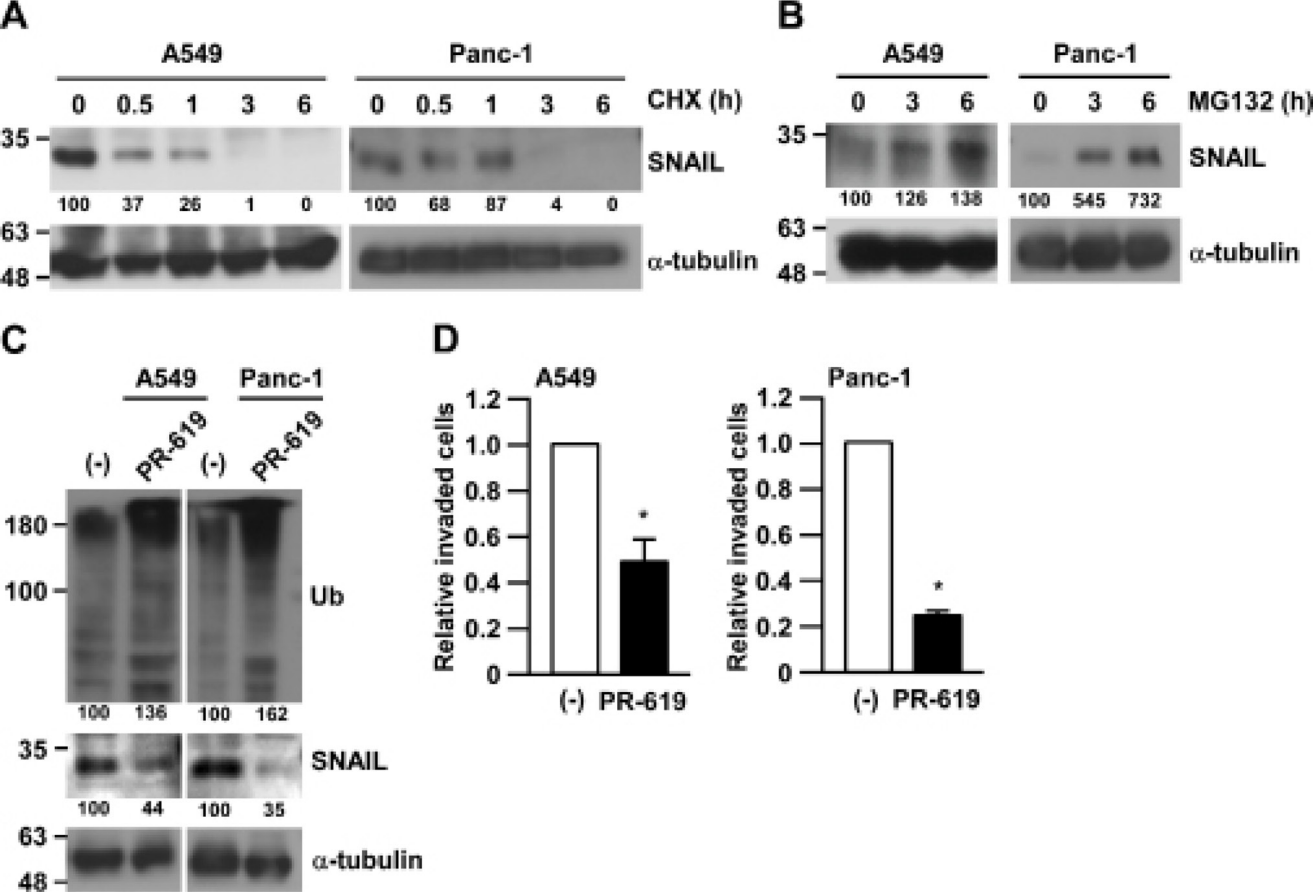
Inhibition of deubiquitinating enzymes suppressed cancer cell invasion (**A**) A549 or Panc-1 cells were treated with 50 µg/ml cycloheximide (CHX) for the indicated times and subjected to Western blotting. The band intensities were measured by ImageJ, normalized to that at 0 h of each cells, and shown below each panel. (**B**) A549 or Panc-1 cells were treated with 10 µM MG132 for the indicated times and subjected to Western blotting. Other conditions were similar to Figure 1A. (**C**, **D**) A549 or Panc-1 cells were treated with 75 μM PR-619 or DMSO for 3 h and subjected to Matrigel invasion assay (C) or Western blotting (D). Invaded cells were counted and normalized with the vehicle control (–). Data are represented as the mean ± S.D. of at least three independent experiments. ^*^P < 0.01 vs DMSO-treated cells by two-tailed student’s *t* test.

SNAIL plays a critical role in migration ability and invasiveness in human lung adenocarcinoma A549 cells and pancreatic ductal adenocarcinoma Panc-1 cells in our previous reports [17]. In addition, the expression of SNAIL has also been known to be regulated by an ubiquitin-proteasome system (UPS) [13]. Indeed, we observed that the reduction or the accumulation of SNAIL protein in human lung adenocarcinoma A549 cells and pancreatic ductal adenocarcinoma Panc-1 cells occurred after treatment with a protein synthesis inhibitor, cycloheximide (CHX) (Figure 1A), or treatment with a proteasome inhibitor, MG132 (Figure 1B), respectively. These results confirmed that SNAIL expression is regulated by UPS and the degradation machinery of SNAIL can be intact in those cancer cell lines.

We then focused on deubiquitinating enzymes (DUBs), which reverses protein ubiquitination and finally stabilizes target proteins. To test whether DUBs are involved in the stability of SNAIL protein in cancer cells, we used a pan-DUB inhibitor, PR-619. While total ubiquitinated protein were accumulated after PR-619 treatment, SNAIL protein was significantly reduced in both A549 and Panc-1 cells (Figure 1C). Along with the reduction of SNAIL expression, the invasiveness of both A549 and Panc-1 cells were largely impaired by PR-619 treatment (Figure 1D). The effects of PR-619 on the invasiveness were stronger than that on the cell growth (Supplementary Figure 1). These results indicate that DUBs regulate SNAIL degradation and cancer cell invasiveness.

### Identification of deubiquitinating enzymes targeting SNAIL

**Figure 2:**
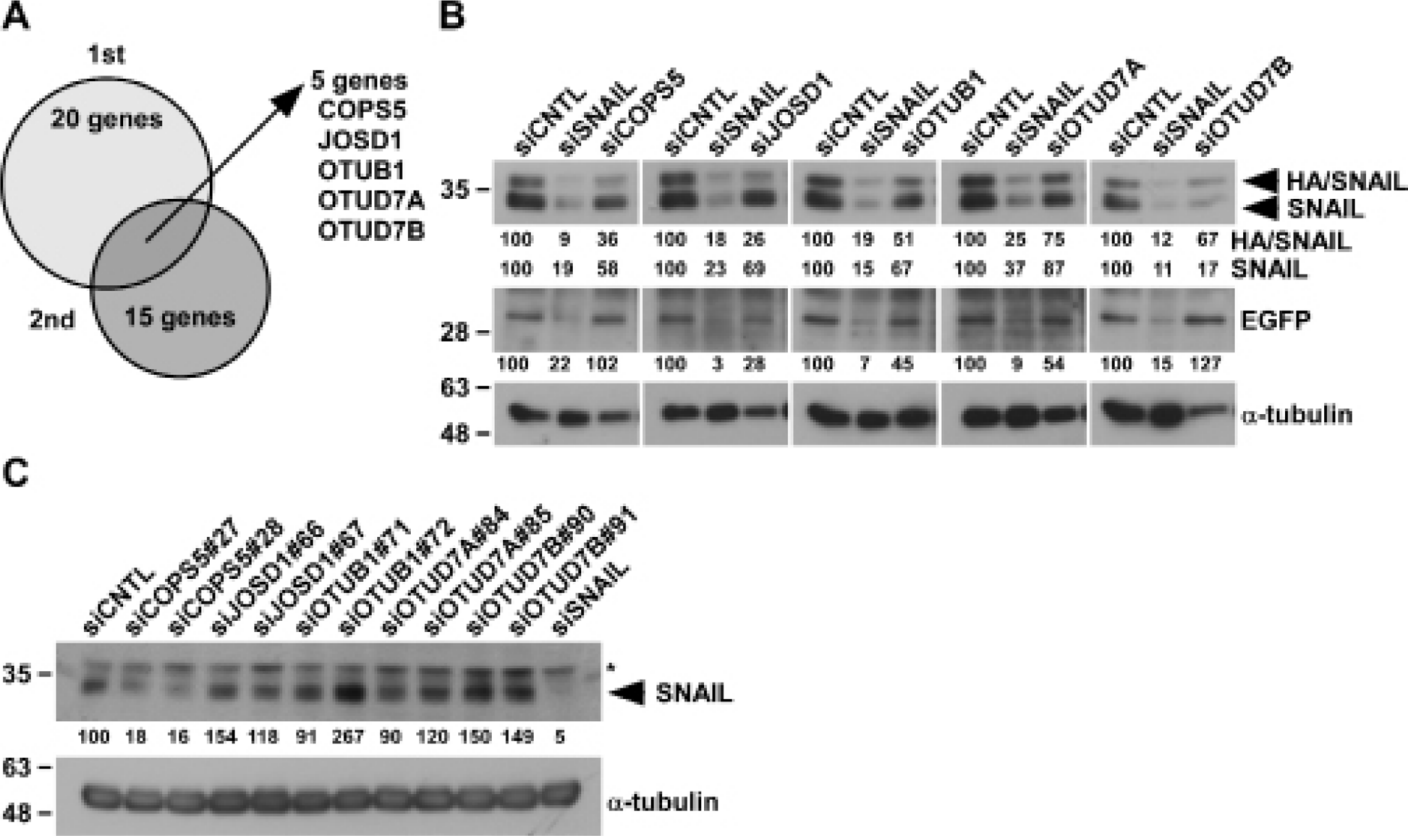
Identification of deubiquitinating enzymes targeting SNAIL (**A**) 12.5 nM siRNAs against each DUB were transfected to HeLa-HA/SNAIL for 96 h and whole cell lysates were subjected to Western blotting. Biological duplicate experiments were performed and 5 candidates were identified as common ones in two experiments. (**B**) The pictures are the results of Western blotting in HeLa-HA/SNAIL cells after knocking-down of SNAIL or 5 candidates, respectively. The band intensities were measured by ImageJ, normalized to that of siCNTL-transfected cells, and shown below each panel. (**C**) A549 cells transfected with the indicated siRNA for 96 h were subjected to Western blotting. ^*^shows non-specific band using anti-SNAIL antibody. Other conditions were similar to Figure 2B.

To identify potential DUBs responsible for SNAIL protein stability in an unbiased manner, we established HA/SNAIL-overexpressing HeLa cells (HeLa-HA/SNAIL) using pIRES2-HA/SNAIL, in which HA/SNAIL and EGFP are transcribed in a single mRNA and separately translated through bicistronic expression. Consequently, we could determine the post-translational regulation of SNAIL by comparing HA/SNAIL expression with EGFP expression. By knocking-down of individual 97 human DUBs by pooled siRNA (four siRNAs per each gene) in HeLa-HA/SNAIL, we found 20 and 15 genes from first screening and second screening, respectively. From these two independent experiments, 5 siRNAs (COPS5, JOSD1, OTUB1, OTUD7A, and OTUD7B) were commonly identified, all of which suppressed both HA/SNAIL and endogenous SNAIL expression compared with control knocked-down cells (siCNTL) (Figure 2A and 2B). We further evaluated the endogenous SNAIL protein in A549 cells by using two individual siRNAs for each gene. Amongst them, COPS5 knockdown could strongly suppress endogenous SNAIL expression (Figure 2C); therefore, we focused on COPS5 in further experiments.

### COPS5 regulates the invasiveness and metastasis of lung cancer cells.

**Figure 3:**
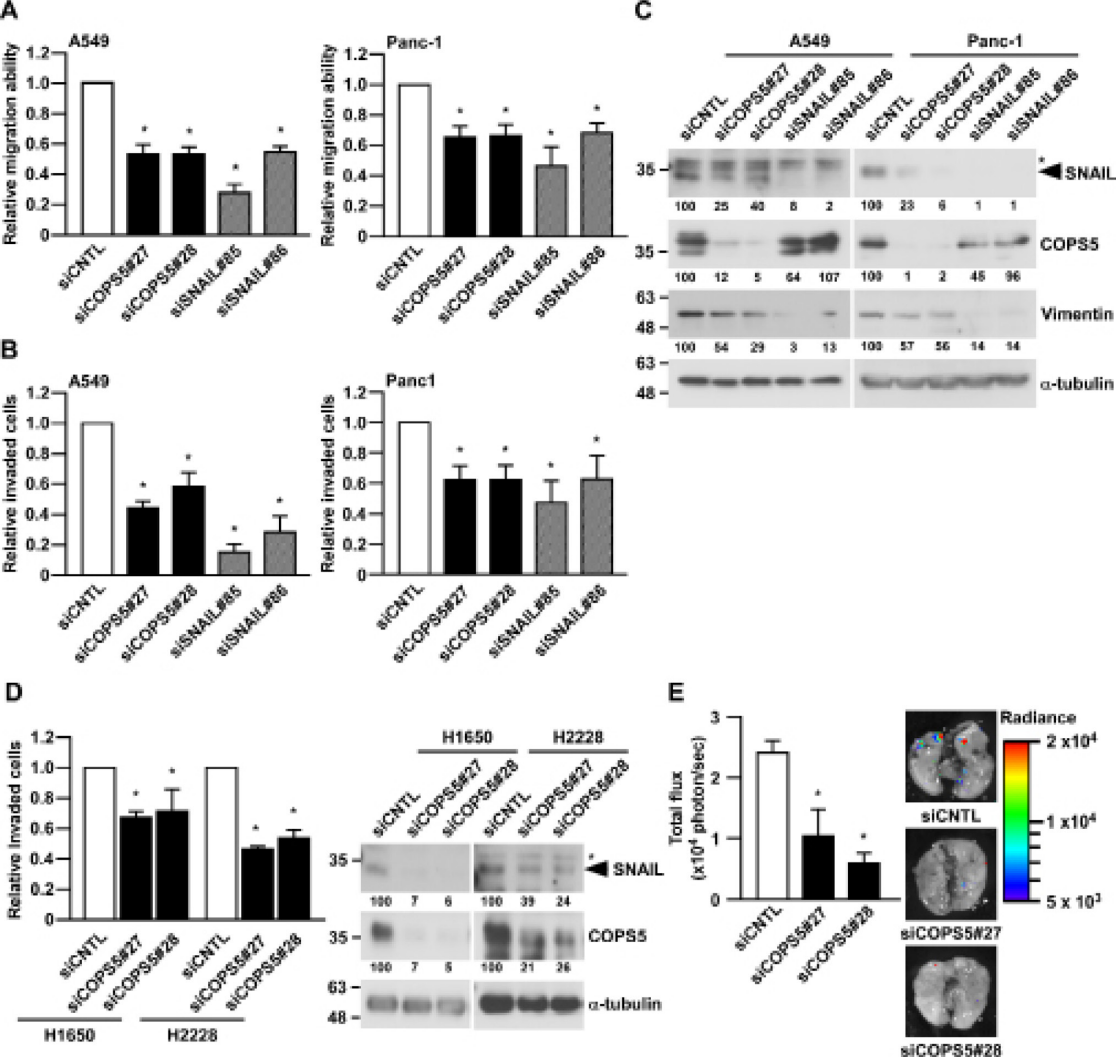
COPS5 regulates the invasiveness and metastasis of lung cancer cells (**A**–**C**) A549 and Panc-1 cells transfected with the indicated siRNA for 96 h were subjected to Wound healing assay (A), Matrigel-invasion assay (B), and Western blotting (C). The band intensities were measured by ImageJ, normalized to that of siCNTL-transfected cells, and shown below each panel. ^*^shows non-specific band using anti-SNAIL antibody. (**D**) Two lung adenocarcinoma cells (H1650 and H2228) transfected with the indicated siRNA for 96 h were subjected to Matrigel-invasion assay (left panel) or Western blotting (right panel). (**E**) A549/Luc2 cells transfected with the indicated siRNA for 96 h were subjected to *In vivo* invasion assay (*n* = 3, each group). Data are represented as the mean ± S.D. of at least three independent experiments. ^*^P <0.01 vs siCNTL-transfected cells by one-way ANOVA followed by the Bonferroni post-hoc test.

To confirm the functional significance of SNAIL suppression by COPS5 knock-down, we next examine the migration ability and invasiveness using A549 cells and Panc-1 cells. Strikingly, the knockdown of COPS5 significantly decreased both the migratory ability and invasiveness of both A549 and Panc-1 cell lines (Figure 3A and 3B), in concert with the reduction of SNAIL and its downstream target protein, Vimentin (Figure 3C) [18]. Because both A549 and Panc-1 are KRAS mutant cancer cell lines, we next checked the effects of COPS5 knock-down in H1650 or H2228 lung adenocarcinoma cell lines, which has an EGFR mutation or EML4-ALK fusion, respectively. As shown in Figure 3D, their invasiveness were significantly suppressed by knocking-down of COPS5 in both H1650 and H2228 in concert with SNAIL reduction, suggesting the important role of COPS5 in the invasiveness of lung cancers independent of their specific oncogenic mutations.

Next, we tested the functional impact of COPS5 on lung cancer metastasis *in vivo* by using A549 cells expressing the firefly luciferase gene (A549/Luc2) [17]. Consistent with the *in vitro* results, the knockdown of COPS5 in A549/Luc2 cells significantly suppressed the formation of lung metastasis compared to the control knockdown in A549/Luc2 cells (Figure 3E). We also detected SNAIL reduction after the knockdown of COPS5 in A549/Luc2 cells (Supplementary Figure 2). These results clearly indicate that COPS5 is involved in the lung metastatic ability of cancer cells even in the physiological condition.

### COPS5 directly regulates SNAIL stability through deubiquitination.

**Figure 4:**
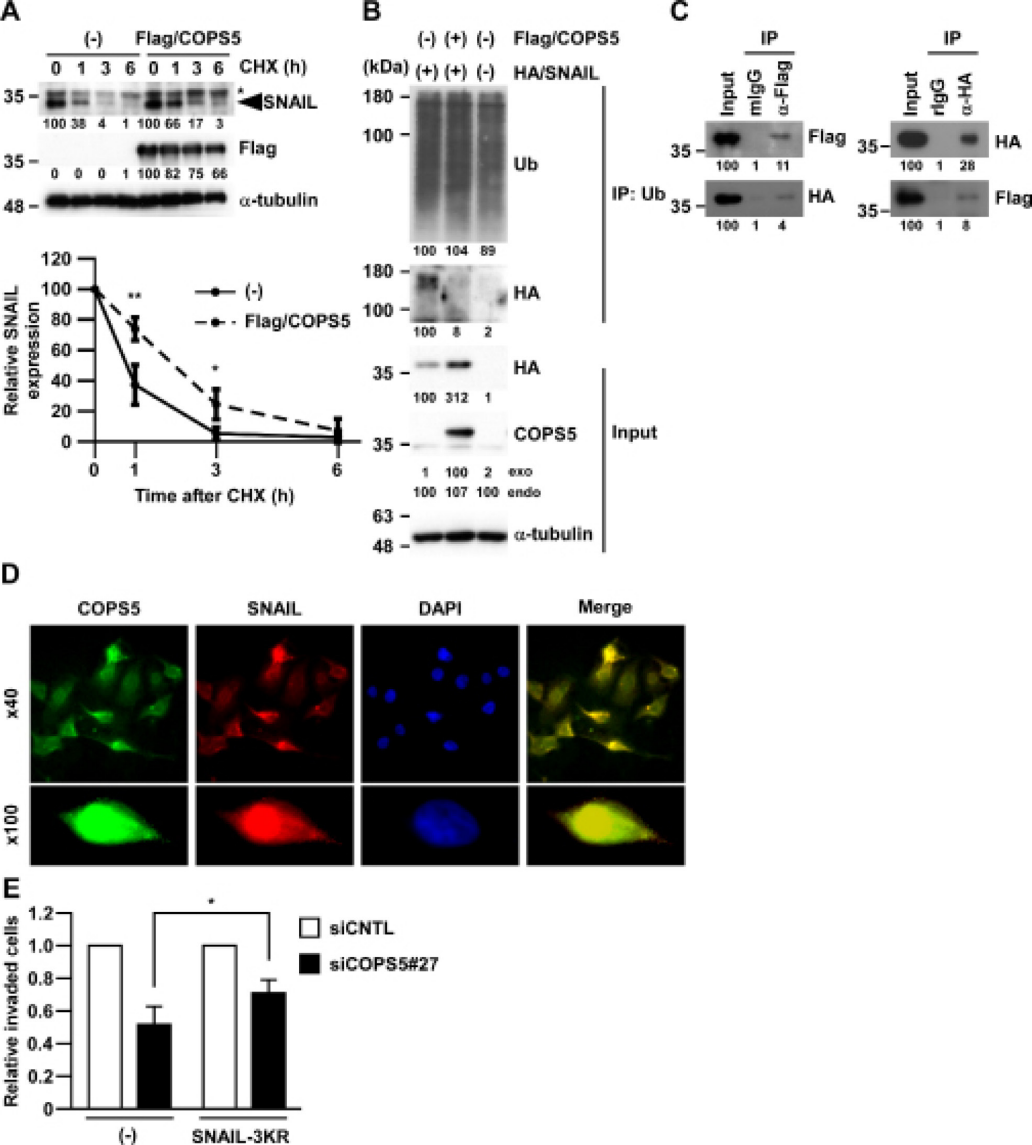
COPS5 directly regulates SNAIL stability through deubiquitination (**A**) A549 cells transfected with Flag-tagged COPS5 (Flag/COPS5) or empty vector (–) were treated with cycloheximide (CHX) for the indicated time and subjected to Western blotting (upper panels). ^*^shows non-specific band using anti-SNAIL antibody. The band intensities in biological triplicated experiments were measured by ImageJ and normalized to that at 0 h of each transfected cells (lower panel). Data are represented as the mean ± S.D. of three independent experiments. ^**^P < 0.01 or ^*^P < 0.05 vs non-transfected cells by two-way ANOVA followed by the Bonferroni post-hoc test. (**B**) A549 cells were transfected with either Flag/COPS5 or HA/SNAIL for 48 h. After immunoprecipitation of the ubiquitinated protein, the cell lysates were subjected to Western blotting. The band intensities were measured by ImageJ and normalized to that of each control. (**C**) A549 cells were transfected with both Flag/COPS5 and HA/SNAIL for 48 h. After immunoprecipitation using anti-Flag antibody (left panel) or anti-HA antibody (right panel), the cell lysates were subjected to Western blotting. Other conditions were similar to Figure 4B. (**D**) A549 cells were treated by MG132 for 3 h and stained by SNAIL (red), COPS5 (green), and DAPI (blue), respectively. The fluorescent images are shown as pictures under a microscope at ×40 (upper panels) or at ×100 (lower panels). (**E**) A549 cells transfected with each siRNA (96 h) and HA/SNAIL-3KR (48 h) were subjected to Matrigel Invasion assay. Data are represented as the mean ± S.D. of at least three independent experiments. ^*^P < 0.01 vs siCNTL-transfected cells by two-way ANOVA followed by the Bonferroni post-hoc test.

To directly address whether COPS5 stabilizes SNAIL protein, A549 cells overexpressing Flag-tagged COPS5 (Flag/COPS5) were treated with CHX, an inhibitor of protein synthesis. As shown in Figure 4A, the half-life of SNAIL protein upon CHX treatment was prolonged by the overexpression of Flag/COPS5 compared with the vector control. To further investigate whether COPS5 functions as a DUB for regulating SNAIL expression, we examined the effects of COPS5 overexpression on SNAIL ubiquitination in A549 cells by immunoprecipitation assay. While the ubiquitination of HA/SNAIL was observed, it was significantly decreased by co-expressing Flag/COPS5 in A549 cells (Figure 4B). Furthermore, co-immunoprecipitation between Flag/COPS5 and HA/SNAIL in A549 cells (Figure 4C) and co-localization of COPS5 with SNAIL in immunofluorescence staining (Figure 4D and Supplementary Figure 3) supported the direct association of COPS5 and SNAIL. Collectively, these results indicate that COPS5 can be a bona fide deubiquitinating enzyme of SNAIL protein.

To investigate the significance of SNAIL deubiquitination by COPS5 in cancer metastasis, we introduced the mutant forms of SNAIL protein (K98R/K137R/K146R, referred to as SNAIL-3KR) in A549 cells, which lacks three ubiquitination sites [13] and therefore increased protein stability (data not shown). As shown in Figure 4E, SNAIL-3KR partially, but statistically significantly, rescued the impaired invasion of A549 cells by COPS5 knock-down. These results indicate the role of endogenous COPS5 in SNAIL-dependent cancer cell invasion through the deubiquitinating activity of COPS5.

**Figure 5:**
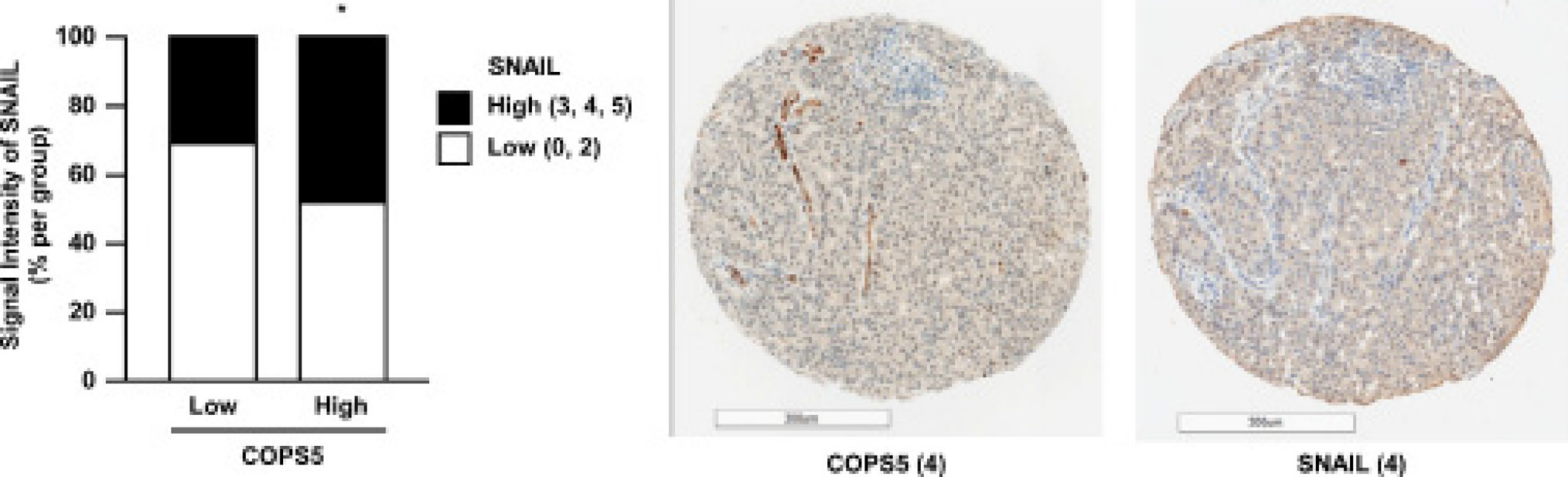
COPS5 expression is positively correlated with SNAIL expression in lung adenocarcinoma tissues A lung adenocarcinoma tissue microarray, including 200 cores, was adopted for immunohistochemical staining using primary antibodies against SNAIL and COPS5. Nuclear and/or cytoplasmic staining for SNAIL or COPS5 was considered to indicate positivity. Image capture and evaluation were performed in a masked manner by two independent researchers. Among 200 cores containing lung adenocarcinoma tissues, 168 cores were scored by two independent researchers. Finally, 160 cores were classified into same groups by two researchers and used as available cores. The sum of the distribution score (0–2) and the intensity score (0–3) was converted into negative (DS + IS; 0 or 2) and positive (DS + IS; 3–5). The correlation between COPS5 and SNAIL expression was evaluated by Chi-square test among the patient subgroups. ^*^P < 0.05 was considered statistically significant. Typical staining images of lung cancer tissues are shown. Scale bar, 300 μm.

Finally, to explore the clinical relevance of the present findings, we examined the expression of COPS5 and SNAIL in the tissue microarray containing 160 lung adenocarcinomas (Figure 5 and Supplementary Figure 4). In alignment with our presented findings in the cell lines, the higher levels of COPS5 expression were correlated with SNAIL expression in the lung adenocarcinoma tissue samples as determined by the immunohistochemistry scoring. Collectively, these results strongly support the association between COPS5 and SNAIL expression in the clinical cancer samples likely through the deubiquitinating activity of COPS5.

## DISCUSSION

The significance of SNAIL functions is not only known in cancer metastasis but also in epithelial-to-mesenchymal transition (EMT) [3], cancer stem cell properties [5], immune evasion [6], and cancer metabolism [8]. The SNAIL protein stability, cellular localization, and activity are known to be regulated by post-translational modifications such as phosphorylation, acetylation, and ubiquitination. Although there are several reports regarding the ubiquitin ligases of SNAIL, little is known about the deubiquitinating enzyme of SNAIL, which reverses its ubiquitination and therefore stabilizes the protein expression of SNAIL. Here, we identified COPS5 as a deubiquitinating enzyme (DUB) of SNAIL to regulate the metastatic ability of lung cancer cells. The positive correlation between SNAIL and COPS5 expression was also relevant in the tissue microarray analysis of human lung adenocarcinoma panel. Importantly, COPS5 knock-down did induce epithelial-like phenotype in A549 cells, as seen in the reduction of vimentin expression (Figure 3C) and the morphological changes from spindle-like shape to cobblestone-like shape (data not shown). These results clearly show the importance of COPS5 as a deubiquitinating enzyme of SNAIL in the metastatic progression of cancer cells.

COPS5, also known as CSN5 or JAB1, is one of the subunits of COP9 signalosome, which is a highly conserved protein complex between the species [19]. COPS5 is a multifunctional protein that plays an important role in the regulation of chromosome instability, DNA damage, and cell cycle [20] targeting K48 and K63-linked polyubiquitination [21]. Dysregulation of COPS5 activity has been shown to contribute to oncogenesis through its function in cell proliferation [22], cell cycle progression [23], and cell chemo-/radio-resistance [21, 24], that are mediated by the various target molecules such as MDM2, p27, HIF-1α, and AP-1 [25]. Recently, the relevance of COPS5 amplification and overexpression in the tamoxifen-resistance of ERα-positive breast cancer was also reported [21]. Although little is known regarding the involvement of COPS5 in cancer metastasis, the role of COPS5 in the invasion of human colorectal cancer cell lines was previously studied [26]. In addition to its direct contribution in cancer cell invasion, COPS5 is known to regulate TGF-β signaling by binding to Smad7 [27], which is the important signaling pathway for both metastasis and EMT. The overexpression of COPS5 promoted the degradation Smad7, and is therefore considered to increase Smad2 phosphorylation to enhance TGF-β-induced transcriptional activity. Although the potential involvement of Smad7 in COPS5-dependent metastasis regulation still needs to be determined, we observed the sustained SNAIL mRNA expression (data not shown) even after COPS5 knock-down, supporting the post-translational SNAIL regulation by COPS5. In addition, COPS5 is also known as a common component of various smaller complexes containing some other subunits of COP9 signalosome [28] such as CSN2 and CSN6. While CSN2 has been shown to regulate SNAIL stability through inhibiting its phosphorylation by GSK-3β [29], CSN6 has been shown to be overexpressed in colorectal cancer and involved in its development [30]. COPS5 has also reported to suppress the SNAIL ubiquitination by inhibiting the binding of SNAIL with β-Trcp, an E3 ligase [29]. As we observed that the ectopic expression of non-ubiquitinated mutant SNAIL (SNAIL-3KR) enhanced the impaired invasion of COPS5 knock-down A549 cells (Figure 4E), the ubiquitination of SNAIL at least in part affects SNAIL function and the metastatic potential of cancer cells. Moreover, only partial rescue by the mutant SNAIL in our study implies the sustained SNAIL ubiquitination and degradation through additional ubiquitination sites such as K85 and K234 [31]. Although the effects of COPS5 knock-down in SNAIL expression was most predominant amongst the candidates in lung adenocarcinomas, the other four DUBs (JOSD1, OTUB1, OTUD7A, and OTUD7B) could potentially regulate SNAIL expression in other cancer such as cervical cancers (Figure 2). Indeed, OTUB1 has been reported to promote metastasis by facilitating EMT [32]. Nevertheless, COPS5, as well as COP9 signalosome, can be an attractive molecular target for cancer metastasis through the regulation of the SNAIL pathway.

Regarding the positive correlation between COPS5 and SNAIL expression in lung adenocarcinoma tissues (Figure 5), we also found the correlation between high COPS5 expression and poor survival of lung adenocarcinoma patients by analyzing the GEO datasets (GSE13213 and GSE31210) [33, 34] in Prognoscan (Supplementary Figure 4) [35]; therefore, COPS5 could be a prognostic marker for lung cancer patients. Strikingly, it is demonstrated that non-cancerous lung tissues showed significantly lower COPS5 expression than those in lung cancer tissues [36]. Moreover, these clinical data, which do not specify any oncogenic mutation, and our invasion experiments using some lung cancer cells with different mutation including KRAS (A549), EGFR (H1650), and EML4-ALK (H2228) (Figure 3D) imply that the target therapy for COPS5 may be applicable to the lung cancer patients even with different mutation types. Besides lung cancer [37], COPS5 is overexpressed in many other types of cancer, including breast [38], pancreatic [39], and hepatocellular carcinoma [40]. Not only its overexpression, but also COPS5 gene amplification has been recently reported in ERα-positive breast cancer, supporting that COPS5 might be an oncogene in some cancers [21]. The important role of SNAIL in cancer progression and metastasis is also relevant to many types of cancer other than lung cancer [3]. Collectively, our present study sheds light on the COPS5 deubiquitinating enzyme to control SNAIL-dependent metastasis and progression of COPS5-overexpressing cancers, and further implicates the new therapeutic strategy of cancer by targeting COPS5.

## MATERIALS AND METHODS

### Reagents and plasmid preparation

A protein synthesis inhibitor, cycloheximide, was purchased from Cayman Chemical (Ann Arbor, MI, USA). A proteasome inhibitor (MG132) and a non-selective, reversible inhibitor of deubiquitination (PR-619) were purchased from Merck Millipore (Billerica, MA, USA).

Human SNAIL or COPS5 cDNA was amplified by RT-PCR from cDNA derived from A549 cells and inserted into pIRES2-EGFP (Takara, Shiga, Japan) and pcDNA3.1-HAHA vector (a kind gift from David E. Fisher, MGH, Boston, MA, USA), or p3xFLAG-CMV7.1 vector, respectively. Point mutations of K98R, K137R, and K148R were introduced by following the protocol of the QuickChange site-directed mutagenesis kit (Stratagene, La Jolla, CA, USA), named pcDNA3.1-HA/SNAIL-3KR.

### Cell cultures

Human cervical cancer cell lines (HeLa), human lung adenocarcinoma cell lines, (A549), and human pancreatic ductal adenocarcinoma cell lines (Panc-1) were obtained from the American Type Culture Collection (ATCC, Rockville, TX, USA). Two human lung adenocarcinoma cell lines (H1650 and H2228) were kindly gifted by Dr. Yano, S. (Kanazawa University, Ishikawa, Japan). All cell lines were maintained in RPMI1640, containing 10% FBS, 1 mM L-glutamine, and antibiotics (100 units/mL penicillin and 100 mg/mL streptomycin) in a humidified atmosphere of 95% air and 5% CO_2_ at 37° C.

To establish HeLa cells stably expressing HA/SNAIL, HeLa cells were transfected with pIRES2-HA/SNAIL, selected, and cloned in growth medium containing 1 mg/mL G418. A549/Luc2 cells were previously established [17].

For siRNA screening, 12.5 nM Dharmacon siGENOME SMARTpool siRNA Library (Human Deubiquitinating Enzymes) and siGENOME Non-targeting siRNA Pool #2 (Thermo Fisher Scientific, Rockford, IL, USA) were transfected in HA/SNAIL-overexpressing HeLa cells (HeLa-HA/SNAIL) using Lipofectamine RNAiMAX reagent (Thermo Fisher Scientific). For knocking down experiments, Silencer Select Negative Control #1, or Silencer Select siRNAs against COPS5 (s21627 and s21628), JOSD1 (s19266 and s19267), OTUD7A (s46284 and s46285), and OTUD7B (s32490 and s32491) (Thermo Fisher Scientific) were transfected in A549 and Panc-1 cells using Lipofectamine RNAiMAX reagent. The transfected cells were subjected to a Matrigel invasion assay, Wound healing assay, or Western blotting after 96 h.

For transient transfection, expression vectors were co-transfected into A549 cells using Lipofectamine 2000 reagent (Thermo Fisher Scientific) and the transient transfected cells were subjected to a Matrigel invasion assay or Western blotting after 24 h.

### Western blotting and immunoprecipitation assay

Whole cell lysates were prepared as described previously [17]. Primary antibodies used were SNAIL, COPS5, Vimentin from Cell Signaling Technology (Beverly, MA, USA), α-tubulin from Santa Cruz Biotechnology (Santa Cruz, CA, USA), anti-FLAG antibody (Sigma-Aldrich, St. Louis, MO, USA), and anti-HA antibody from Roche (Indianapolis, IN, USA). The ubiquitination of SNAIL was assessed by Ubiquitin Enrichment Kit (Thermo Fisher Scientific) according to the manufacturer’s instructions. The immunoprecipitation lysates were subjected to Western blotting. The band intensities were measured by ImageJ and normalized to that of each control lane.

### Wound healing assay

Wound healing assay was performed as described previously [41]. Briefly, A549 and Panc-1 cells (1 × 10^5^ cells/well) were seeded into a 24-well plate and treated 12.5 nM siRNA transfection. After 96 h siRNA transfection, confluent cells were scratched using pipette tips. After scratching, the scratched width was measured at 0 h and 48 h.

### Matrigel invasion assay

Matrigel invasion assay was performed as described previously [17]. Briefly, membrane filters (Whatman, Maidstone, UK) were attached to Transwell chambers (Costar, Cambridge, MA, USA) and the lower surface was pre-coated with 1.25 µg fibronectin (Iwaki, Tokyo, Japan) and the upper surface was pre-coated with 5 µg Matrigel (BD Biosciences, Franklin Lakes, NJ, USA). Pre-treated cells (3 × 10^4^ cells/100 µl in RPMI 1640 Medium with 0.1% bovine serum albumin) were added to the upper compartment of the chamber. After incubation for 6 h, the invaded cells were stained by hematoxylin and eosin and counted manually under a microscope at 50x magnification.

### Experimental lung metastasis experiment

Experimental lung metastasis experiments were performed as described previously [17, 42]. C.B-17/lcrHsd-Prkdcscid mice were purchased from Japan SLC (Hamamatsu, Japan). All experiments were approved and performed according to the guidelines of the Care and Use of Laboratory Animals of the University of Toyama. Cells were inoculated intravenously (2 × 10^6^ cells/200 µL PBS/mouse) into mice and 200 µL of luciferin (1.5 mg/mL [VivoGlo; Promega, Madison, WI, USA]) was intraperitoneally injected into mice at 24 h after the tumor inoculation. After 20 min, the lungs were removed to subject bioluminescent assay by using an *in vivo* imaging system (IVIS Lumina II, Caliper Life Sciences, Hopkinton, MA, USA). The data are presented as the mean luminescence ± SEM.

### Immunofluorescence staining

A549 cells were treated with 10 µM MG132 for 6 h. After fixation using 4% formaldehyde at room temperature for 15 min, the cells were incubated overnight at 4**°** C with goat polyclonal anti-SNAIL (1:200; Abcam, MA, USA) and rabbit polyclonal anti-Jab1 (COPS5) protein (1:200, Abcam). The cells were then stained with Alexa Fluor 555-anti-goat IgG (1:500, Thermo Fisher Scientific) and Alexa Fluor 488-anti-rabbit IgG (1:500, Thermo Fisher Scientific) at room temperature for 90 min and mounted using VECTASHIELD mounting media with DAPI (Vector Laboratories, Burlingame, CA).

### Immunohistochemical staining

A lung cancer tissue microarray, which consists of 353 cores, was obtained from Toyama University Hospital (Toyama, Japan). Detailed clinical and pathologic information, including patient demographics, smoking history, and overall survival, was available for most patients in the lung cancer tissue microarray. This study was approved by the Institutional Review Boards of University of Toyama. Immunohistochemical staining was performed using BOND-III Fully Automated IHC (Leica Biosystems) at Pathology Institute Corporation (Toyama, Japan). Sections were cut and deparaffinized through graded alcohol and xylene. After antigen retrieval using BOND ER1 buffer, the sections were incubated for 30 minutes at room temperature with goat polyclonal anti-SNAIL (diluted at 1:200; Abcam, MA, USA) and rabbit polyclonal anti-Jab1 (COPS5) protein (1:200, Abcam). After incubation with rabbit polyclonal anti-goat serum (1:1000, Bethyl Laboratories, Montgomery, TX, USA), the sections were incubated for 30 minutes at room temperature with BOND Polymer Refine Detection System-HRP (Leica). Then, endogeneous peroxidase was blocked with 3% hydrogen peroxidase for 5 minutes, visualized with DAB, and counterstained with hematoxylin, dehydrated, cleared, and mounted with resinous mounting medium. Nuclear and/or cytoplasmic staining for SNAIL or COPS5 was considered to indicate positivity. Image capture and evaluation was performed in a masked manner. We scored immunohistochemical staining as described previously [43, 44]. Briefly, the distribution score (DS), which reflects the distribution of the positive signal among tumor cells, was scored as 0 (0%), 1 (1%–50%), or 2 (51%–100%) to reflect the percentage of positive tumor cells among whole tumor cells seen in the same tissue disk. The intensity score (IS), intensity of the signal, was scored as 0 (no signal), 1 (weak), 2 (moderate), or 3 (marked). The sum of DS and IS was converted into negative (DS + IS; 0 or 2) and positive (DS + IS; 3–5) [43, 45]. Among 200 cores containing lung adenocarcinoma tissues, 168 cores were scored by two independent researchers. Finally, 160 cores were classified into same groups by two researchers and used as available cores.

### Statistical analysis

Statistical significance was calculated using Graphpad Prism software (GraphPad Software, Inc, San Diego, CA). More than three means were composed using one-way ANOVA with the Bonferroni correction, and two means were composed using unpaired Student’s *t*-test. The correlation between COPS5 and SNAIL expression in immunohistochemical stainings was evaluated by Chi-square test among the patient subgroups. *P* < 0.05 was considered statistically significant.

## SUPPLEMENTARY MATERIALS FIGURES


