# Supplementary material for: COP9 signalosome subunit 5 regulates cancer metastasis by deubiquitinating SNAIL
Source: Oncotarget. 2018 Apr 17;9(29):20670–80. doi: 10.18632/oncotarget.25060 (PMC5945527; doi:10.18632/oncotarget.25060)
Supplement: Supplementary file 1 [file oncotarget-09-20670-s001.pdf]

COP9 signalosome subunit 5 regulates cancer metastasis by deubiquitinating SNAIL

SUPPLEMENTARY MATERIALS

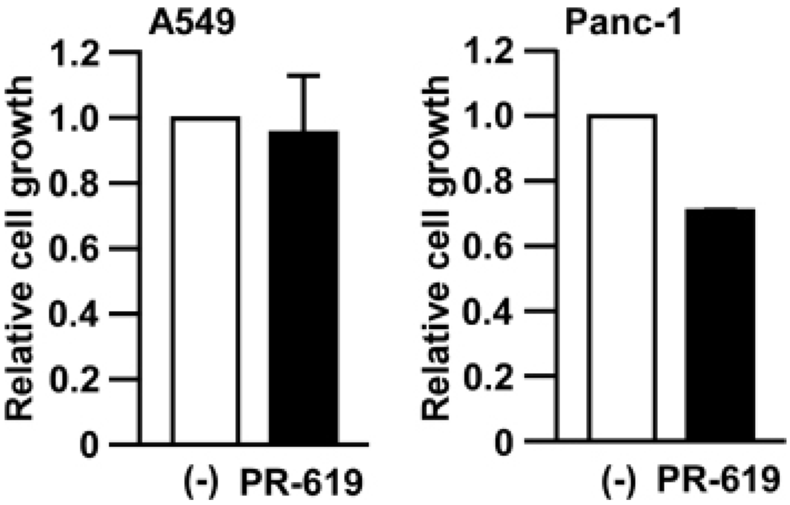

Supplementary Figure 1: PR-619 did not strongly suppress the cell growth in A549 and Panc-1. A549 or Panc-1 cells were treated with 75 µM PR-619 or DMSO for 3 h. After washing out PR-619, the cells were incubated for 6 h and subjected to CellTiter-Glo assay. The relative cell growth was normalized to vehicle control by CellTiter-Glo assay.

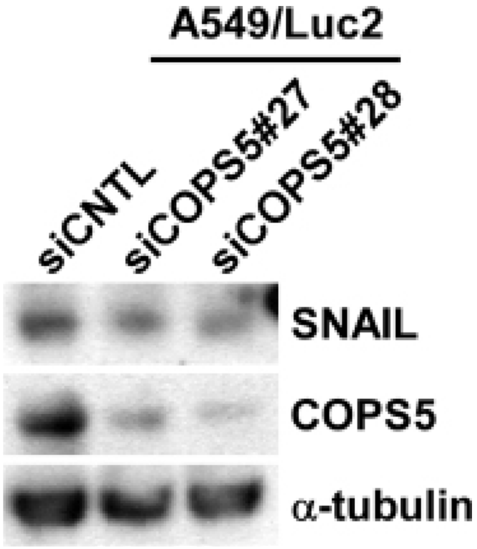

Supplementary Figure 2: SNAIL expression was reduced by COPS5 knockdown in A549/Luc2 cells. A549/Luc2 cells transfected with the indicated siRNA for 96 h were subjected to Western blotting.

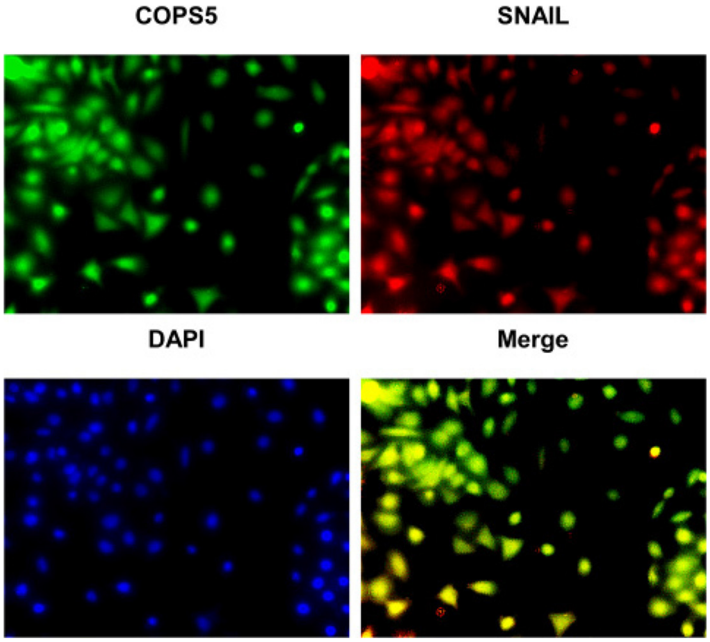

Supplementary Figure 3: COPS5 co-localized with SNAIL in A549 cells. A549 cells were treated by MG132 for 3 h and stained by SNAIL (red), COPS5 (green), and DAPI (blue), respectively. The fluorescent images are shown as pictures under a microscope at x40 (upper panels).

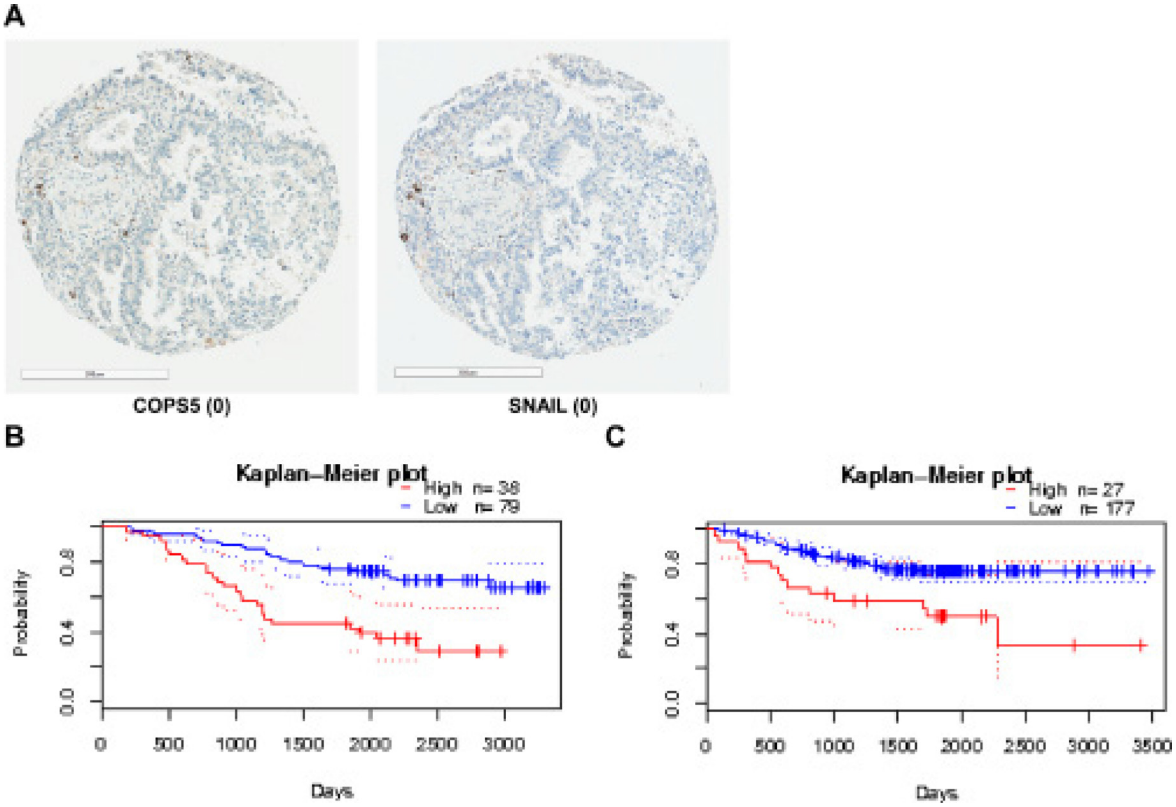

Supplementary Figure 4: High COPS5 expression is correlated with poor prognosis in lung adenocarcinomas. (**A**) Typical staining images of lung cancer tissues in negative group (DS+IS; 0) are shown. (**B**, **C**) Representative results of COPS5 expression in lung adenocarcinoma cases, GSE13213 (B) or GSE31210 (C), analyzed by Prognoscan . Cases were arranged by the expression of COPS5 (A_23_P71419 or 201652_at). Kaplan–Meier curves of overall survival (B) or relapse-free survival (C) for high (red) and low (blue) expression groups polarized the optimal cutoff point are plotted.
